# Remarks on the Use of Spirit of Turpentine in Incarcerated Hernia

**Published:** 1830-05

**Authors:** C. B. Hamilton

**Affiliations:** late Surgeon of the Marine Hospital at Washington City.


### TURPENTINE IN HERNIA.

*Remarks on the Use of Spirit of Turpentine in Incarcerated Hernia.* By C. B. Hamilton, late Surgeon of the Marine Hospital at Washington City.

In the last Number of this Journal, I have noticed a paper by Professor Sewall,* on the use of the spirit of turpentine internally as a remedy in incarcerated hernia. In his concluding paragraph the Professor observes, "it requires the experience derived from many cases to entitle a new remedy to confidence;" and it may be added, that a proper application of a remedy to those diseased conditions of the system in which, from analogy and reason confirmed by experience, it is found to prove beneficial, is equally necessary to sustain that confidence when it is acquired.

I have for many years used the spirit of turpentine in incarcerated hernia, without being aware that it was a new remedy, and without its being in every instance successful; for in one case in which I employed it as a dernier resort,

* The paper referred to is contained in our Number for March last. —Editor *Lond. Med. and Phys. Journ.*

upon the patient's positively refusing to submit to an operation, no mitigation of the disease, but rather an aggravation of suffering, resulted from its exhibition. This was a case of omental inguinal hernia, and the patient died with all the symptoms of supervening mortification. That the hernial sac contained a portion of omentum only, I inferred from the bowels yielding to the operation of purgative medicine, which could not have been the case had a portion of the intestinal tube been shut up by the stricture: the stricture in this case was in the tendon forming the ring, and therefore beyond the immediate influence of a remedy applied to the stomach. Among the earlier recollections of my boyhood, is the use of the spirit of turpentine in spasmodic or flatulent colic; and a case that came under my observation when about ten years of age served to fix its use in this disease indelibly on my memory. This was a case in which an uterine inflammation succeeding to concealed abortion, in the person of a servant girl, was mistaken by her mistress for colic, and the turpentine administered with the most melancholy effect.

Being called to a case, some years ago, of strangulated scrotal hernia, of but a few hours' standing, which, from the great distention of the strangulated bowel by flatus and excrement, resisted all my efforts at reduction by taxis, I was naturally led to speculate upon the cause of so great and sudden an accumulation in the gut. It struck me that if the occluding stricture existed in the abdominal ring, it must necessarily act alike upon the descending and ascending portions of the intestine, and that, of course, nothing could be derived to the incarcerated portion from that within the abdomen, to give it the volume it possessed. It therefore occurred to me that the descending portion of the tube was free, and that the distention was caused by a stricture taking place in the muscular fibres of the ascending portion, and arresting the passage of the contents of the bowels brought down by the peristaltic motion. Considering this state of things to differ in no particular from that which takes place in spasmodic colic, I at once resolved to make trial of the turpentine, the good effects of which I had so often witnessed in the latter disease, and it succeeded beyond my most sanguine anticipations. In a few moments the contents of the strangulated bowel were spontaneously removed, and the intestine restored to the abdominal cavity by taxis, with perfect ease.

About twelve months since, I was called to a coloured man, the property of John Addison, Esq. of this district.

On my arrival, I was informed by his master that he had been for many years afflicted with scrotal hernia; that he had been in the habit of reducing it himself; that a few hours before he had been seized with severe pain in the part, and that the rupture now resisted his usual efforts to reduce it. On examining the patient, I found the scrotum so enormously enlarged that no trace of a penis could be seen; the integuments were cold to the touch, and the swelling elastic. The patient informed me that, a short time before the attack of pain, he had eaten a quantity of unripe fruit, and ascribed his situation to that cause. Without making any attempt at reduction, I inquired if there was any spirit of turpentine in the house, and fortunately about the half of a common-sized wineglassful was produced, which I immediately administered. The relief was instantaneous: the spasm was removed; the air and feces, by the elastic pressure of the intestine, were carried upwards with a gurgling sound into its continuous portion within the abdomen, and, in five minutes after, the patient with his own hand reduced the rupture.

I have made these remarks for the purpose of directing the attention of practitioners to what I consider to be the only condition of the parts, (which, by the way, might, I conceive, be properly termed a scrotal colic,) in which the turpentine proves an invaluable remedy, and to express my opinion of the impropriety of administering it in those cases where the obstruction arises from a stricture of the tendon forming the abdominal ring, or from chronic enlargement of the incarcerated viscera.*

---

### PULMONARY CONSUMPTION.

### *Observations on Pulmonary Consumption.*

* American Journal of Med. Sciences.

